# Large-scale copy number variants (CNVs): Distribution in normal subjects and FISH/real-time qPCR analysis

**DOI:** 10.1186/1471-2164-8-167

**Published:** 2007-06-12

**Authors:** Ying Qiao, Xudong Liu, Chansonette Harvard, Sarah L Nolin, W Ted Brown, Maryam Koochek, Jeanette JA Holden, ME Suzanne Lewis, Evica Rajcan-Separovic

**Affiliations:** 1 Department of Pathology, UBC, Children's and Women's Health Centre of BC, 4480 Oak Street, Vancouver, V6H 3V4, British Columbia, Canada; 2 Department of Medical Genetics, UBC, Children's and Women's Health Center of BC C234, 4500 Oak Street, Vancouver, V6H 3N1, British Columbia, Canada; 3 Department of Physiology, Queen's University, 191 Portsmouth Avenue, Kingston, K7M 8A6, Ontario, Canada; 4 Autism Research Program, Ongwanada, 191 Portsmouth Avenue, Kingston, K7M 8A6, Ontario, Canada; 5 Autism Spectrum Disorders – Canadian-American Research Consortium; 6 Healthcare Equity for Intellectually Disabled Individuals (HEIDI) Research Program; 7 Department of Human Genetics, New York State Institute for Basic Research in Developmental Disabilities, Staten Island, NY 10314, USA; 8 Department of Psychiatry, Queen's University, 191 Portsmouth Avenue, Kingston, K7M 8A6, Ontario, Canada

## Abstract

**Background:**

Genomic copy number variants (CNVs) involving >1 kb of DNA have recently been found to be widely distributed throughout the human genome. They represent a newly recognized form of DNA variation in normal populations, discovered through screening of the human genome using high-throughput and high resolution methods such as array comparative genomic hybridization (array-CGH). In order to understand their potential significance and to facilitate interpretation of array-CGH findings in constitutional disorders and cancers, we studied 27 normal individuals (9 Caucasian; 9 African American; 9 Hispanic) using commercially available 1 Mb resolution BAC array (Spectral Genomics). A selection of CNVs was further analyzed by FISH and real-time quantitative PCR (RT-qPCR).

**Results:**

A total of 42 different CNVs were detected in 27 normal subjects. Sixteen (38%) were not previously reported. Thirteen of the 42 CNVs (31%) contained 28 genes listed in OMIM. FISH analysis of 6 CNVs (4 previously reported and 2 novel CNVs) in normal subjects resulted in the confirmation of copy number changes for 1 of 2 novel CNVs and 2 of 4 known CNVs. Three CNVs tested by FISH were further validated by RT-qPCR and comparable data were obtained. This included the lack of copy number change by both RT-qPCR and FISH for clone RP11-100C24, one of the most common known copy number variants, as well as confirmation of deletions for clones RP11-89M16 and RP5-1011O17.

**Conclusion:**

We have described 16 novel CNVs in 27 individuals. Further study of a small selection of CNVs indicated concordant and discordant array vs. FISH/RT-qPCR results. Although a large number of CNVs has been reported to date, quantification using independent methods and detailed cellular and/or molecular assessment has been performed on a very small number of CNVs. This information is, however, very much needed as it is currently common practice to consider CNVs reported in normal subjects as benign changes when detected in individuals affected with a variety of developmental disorders.

## Background

There is considerable genomic variability among humans that is not associated with a recognizable clinical phenotype. This variability is evident at both the chromosomal level (as microscopically visible gains or losses of chromosomal bands or regions) [1] and at the single nucleotide level (as single nucleotide polymorphisms (SNPs)) [2]. The gains and losses of sub-microscopic DNA segments larger than 1 kb are termed copy number variants (CNVs) [3]. They represent a newly recognized class of DNA variation, identified as a result of the introduction of comparative genomic hybridization (CGH) array technology that enables the study of variation in the number of copies of specific DNA segments among individuals [4,5]. The widespread presence of CNVs in normal individuals has now been documented using not only array CGH-containing BAC clones [6,7] but also oligonucleotide arrays at high resolution as well SNP data analysis [8-12] and DNA sequence comparisons between individuals [13].

The discovery of CNVs presents investigators with a number of challenges as CNVs complicate the interpretation of array data and efforts to attribute microdeletions and microduplications identified in individuals with constitutional disorders or in cancerous tissues to the disease processes. The role of CNVs in causing or influencing the susceptibility to disease and genome evolution remains largely unknown. A catalogue of published CNVs can be found in the public database [14], and helps to guide the interpretation of array CGH findings. However, the number and identity of polymorphic loci detected in different studies varies considerably [15], likely because of differences across various array platforms, analytical methods and the populations investigated. In addition, copy number differences for many of the CNVs listed in the above database are not confirmed using secondary independent quantification methods (FISH or quantitative DNA methods). Therefore, further work to identify and characterize CNVs in human populations and confirm copy number variability is essential in order to better understand the significance of CNVs and to determine their role in common disorders.

We studied 27 phenotypically normal individuals and detected 42 different sub-microscopic CNVs using the 1 Mb resolution whole genome array-CGH. We used real-time quantitative PCR (RT-qPCR) and FISH to confirm CNVs. The results of these studies are presented.

## Results

### 1. Array-CGH findings of CNVs in normal subjects

By studying 27 phenotypically normal, healthy individuals (17 females and 10 males; 9 African-American, 9 Hispanic and 9 Caucasian) using commercial array-CGH (Spectral Genomics), 42 different CNVs were identified and further classified into 2 subgroups. Group A contains 26 CNVs which were previously reported in the public database [14], either as an identical clone entry or as part of a reported genomic CNV region. The remaining 16 CNVs in Group B of Table 1 represent novel CNVs. In total, 8 CNVs were observed in two or more individuals; the majority of recurrent CNVs (7/8) belonged to group A. Segmental duplications were found in 8 CNVs (6 in Group A and 2 in Group B). Of the 42 CNVs, only 13 were found to contain genes (N = 28) listed in the OMIM database; 9/28 genes were found in one CNV in Group A (RP11-144O23 mapping to 12p13.2). In addition to genes primarily involved in functions of the human immune or sensory systems, signal transduction and metabolism, genes involved in transcription regulation, neurotransmitter transport, cell proliferation and differentiation or development were also identified (Table 1).

**Table 1:** Previously reported (A) and novel (B) CNVs

	**Number**	**Cytoband**	**Clone Name**	**Position (Mb)**	**Ethnic origin**	**Duplication**	**Deletion**	**Overlap with segmental duplications**	**Genes on OMIM list**	**Biological process**
**Group A**	1	1p36.13	RP1-163M9*	16.1	AA	1		Yes		
	2	1p13.3	RP11-259N12	103.4	2C, AA, 2H	3	2	Yes	AMY2A, AMY1A, AMY1B, AMY1C	Glycogen metabolism
	3	1q42.13	RP5-1016N21	229.7	C, H		2	No		
	4	2p12	RP11-345F13	82.8	AA	1		No		
	5	2qter	RP5-1011O17	242.9	C, 2H		3	Yes		
	6	3q26.3	RP11-114M1	178.8	H		1	No		
	7	4q25	RP11-18D18	112.7	H		1	No		
	8	6pter	AL035696.14	0.1	H		1	Yes		
	9	6q12	RP11-80L16	67	AA		1	No		
	10	6q24.1	RP1-69B13	146.7	H		1	No	GRM1	G-protein mediated signaling, neuronal activities
	11	7pter	RP1-164D18	0.1	AA	1		No		
	12	7p21.1	IIID11	18.8	C	1		No		
	13	8p22	RP11-89M16	17.2	C		1	No	SLC7A2, PDGFRL	Amino acid transport, receptor protein tyrosine kinase signaling pathway
	14	10qter	CTC-261B16	135.2	AA	1		No		
	15	11q22.3	RP11-179B7	104.4	AA		1	No		
	16	12p13.2	RP11-144O23	10.9	H	1		No	TAS2R7, TAS2R8, TAS2R9, TAS2R10, PRR4, PRH1, TAS2R13, PRH2, TAS2R14	Taste receptor activity, visual perception, cell adhesion-mediated signaling, immunity and defense
	17	13q21.1	RP11-100C24	56.7	4C, AA, 4H	5	4	No		
	18	14q12	RP11-125A5	27.6	C, 3H	2	2	No		
	19	15q11.2	RP11-80H14	20.4	H	1		No	CYFIP1	Signal transduction, developmental processes
	20	16p11.2	RP11-499D5 *	33.8	H		1	Yes		
	21	16p11.2	RP11-488I20 *	35.6	H	1		No		
	22	16p11.1	RP11-80F22 *	35.7	C, 2H	3		No		
	23	17pter	CTB-68F18	0.1	C	1		No	RPH3AL	Synaptic transmission
	24	17q24.3	RP11-300G13	68.6	H		1	No	KCNJ16, KCNJ2	Cation transport, muscle contraction
	25	19p13.2	RP11-79F15	8.8	C, 2AA, 2H	1	4	Yes	MBD3L1, MUC16	mRNA transcription
	26	19qter	1129-c9	76	C	1		No		
										
**Group B**	1	2q14.3	RP11-270M20	125.3	C	1		No	CNTNAP5	Cell adhension-mediated signaling, synaptic transmission
	2	4q28.1	RP11-77P11	128.2	H		1	No		
	3	4q31.2	RP11-89E4	145.8	H	1		No		
	4	6p24	RP1-103M22	9.5	H		1	No		
	5	7q33	RP11-140I14	134.6	AA	1		No	CNOT4	mRNA transcription regulation
	6	10p12.3	RP11-91D9	19.7	H		1	No		
	7	12pter	RP11-598F7 *	0	C	1		No	SLC6A12	neurotransmitter transport
	8	13q13.1	RP11-87G1	33	AA	1		No		
	9	19q13.43	F21283	63.7	H	1		No	MZF1	regulation of transcription
	10	Xpter	LLNOYCO3M11D2	0	3C, AA	2	2	No		
	11	Xp11.3	RP11-252K10	41.7	H		1	No		
	12	Xp11.21	RP11-266I3 *	53.7	AA	1		Yes		
	13	Xp11.1	ICRFC100G11100	56.1	C	1		No		
	14	Xq21.1	RP11-192B18	84.4	H		1	No		
	15	Xq26.2	CTB-45B24	131.4	H		1	No	PCYT1B, PHF6	Regulation of metabolism and transcription, ovarian follicle development, spermatogenesis
	16	Yq11.2	RP11-91A13 *	17.7	AA	1		Yes		

Note: * Clone showing multiple sites based on e-FISH.

AA: African American; H: Hispanic; C: Caucasian.

The three clones which showed copy number changes most frequently in our subjects were RP11-259N12 (1p13.3), RP11-100C24 (13q21) and RP11-79F15 (19p13.2), and were found in 5, 9 and 5 individuals respectively.

### 2. FISH and RT-qPCR analysis of polymorphic clones

#### a) FISH

**Figure 1:**
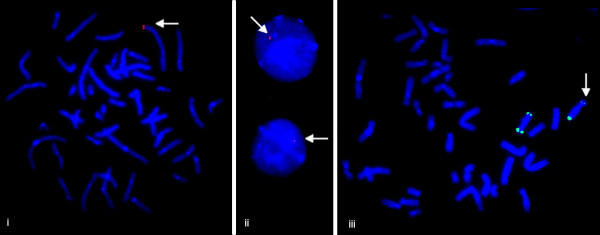
**FISH confirmation of deletions detected by array-CGH**. **i**. Deletion of clone RP5-1011O17 (2q27.3) as demonstrated by a single signal in both metaphase chromosomes (arrowhead) (i) and interphase cells (ii). Partial deletion of clone RP11-89M16 (8p22) is seen as a diminished signal on one of the homologues (iii). A control probe at 8qter, RP11-17M8, was used to eliminate difference in signal intensities due to artifacts. One of the signals on 8p22 was consistently smaller (arrow) than any of the 3 remaining signals on the two chromosome 8 homologues.

In order to establish the cellular copy number pattern of known and novel CNVs, we performed FISH analysis of 6 CNVs (Table 2) in subjects for whom a cell pellet was available. For some clones, copy number changes could be confirmed by FISH, while for others the FISH patterns were discordant with array-CGH results. For example, array analysis of clones RP5-1011O17 at 2q37.3 and RP11-89M16 at 8p22 both showed a deletion upon array analysis, and both were subsequently confirmed by FISH. However, the FISH signal pattern was different for these two clones: clone RP5-1011O17 had a complete loss of one copy, demonstrating a complete deletion while clone RP11-89M16 exhibited a diminished FISH signal on one of the homologs suggestive of a partial deletion (Figure 1).

**Table 2:** List of polymorphic BAC clones used for FISH/qPCR analysis in controls.

**Cytoband**	**Clone name**	**Previously reported**	**Size (kb)**	**Gene(s) contained**	**Overlap with segmental duplications**	**ARRAY**	**FISH**	**qPCR**
2q37.3	RP5-1011O17	Y	21.8	No	yes	deletion	complete loss of one copy (Fig1)	C
13q21.1	RP11-100C24	Y	129.3	No	No	gain	2 copies (Fig3)	NC
						loss	2 copies (Fig2)	NC
14q12	RP11-125A5	Y	186.5	No	No	gain/loss	2 copies (not shown)	NT
2q14.3	RP11-270M20	N	140.4	No	No	gain	2 copies (not shown)	NT
8p22	RP11-89M16	Y	176	MTMR7; SLC7A2; PDGFRL	No	deletion	partial deletion of one copy (Fig 1)	C
12p13.33	RP11-598F7	N	0.5	SLC6A12	No	gain	multiple sites on non-homologous chromosomes (Fig 4)	NT

C = confirmed

NC = not confirmed

NT = not tested

**Figure 2:**
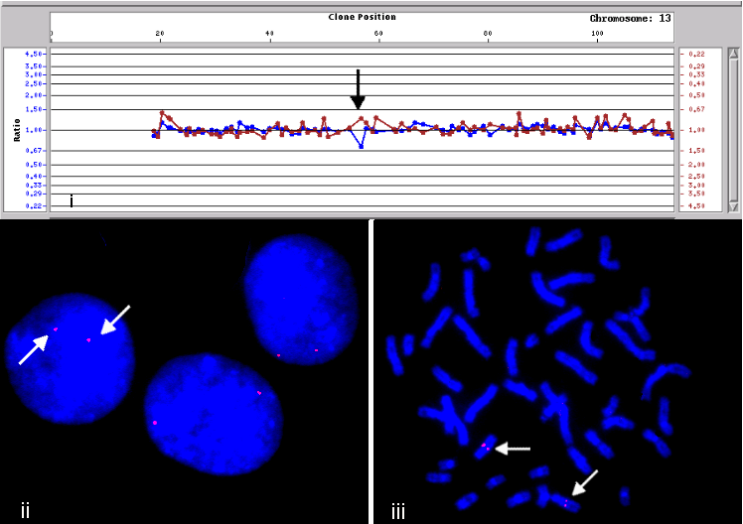
**Array and FISH analysis of BAC clone RP11-100C24 (loss)**. **(i) **The array detected deletion of RP11-100C24 could not be confirmed in interphase (ii) and metaphase cells by FISH (iii). The details of profile interpretation are described in Tyson et al [26]. Briefly, deletion of a clone was considered if the red and the blue array profiles show separation for that clone and the red profile is above the line corresponding to the value of 1. On the other hand, if the blue array profile is above the line corresponding to the value of 1, a gain for the clone is considered.

**Figure 3:**
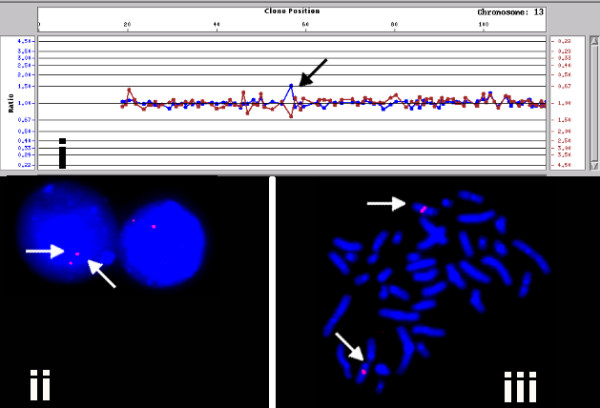
**Array and FISH analysis of BAC clone RP11-100C24 (gain)**. **(i) **The array detected gain of RP11-100C24 could not be confirmed in interphase (ii) and metaphase cells by FISH (iii).

**Figure 4:**
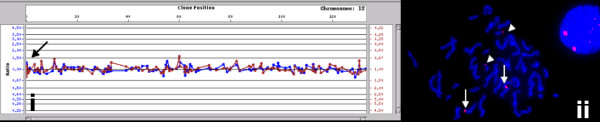
**Duplication of clone RP11-598F7 in a normal subject**. Gain of a terminal clone from 12p on the array is indicated with an arrow (i). FISH probe for this clone hybridizes to multiple non-homologous chromosomes (chromosome 12-arrow; chromosome 20-arrowhead, ii)

Conversely, FISH analysis of clone RP11-100C24 (13q21.1) showed consistent normal signal patterns (2 copies/cell) in multiple subjects regardless of whether the clone was seen as a loss (Figure 2) or gain (Figure 3) on array analysis. Gains of clones RP11-125A5 and RP11-270M20 could not be confirmed by FISH (Table 2). Finally, gain of the clone RP11-598F7 was seen as multiple signals mapping to several chromosomes, demonstrating the presence of homologous sequences within this clone in several regions of the genome (Figure 4). This observation was also confirmed by the *in silico *eFISH simulation tool [16].

#### b) RT-qPCR and FISH comparisons

RT-qPCR analysis was performed for 3/6 clones tested by FISH in order to confirm the array results and to help resolve the array and FISH discrepancies (Table 2). Comparable results between FISH and RT-qPCR were obtained for all 3 clones tested by both methods – i.e. deletions of RP11-89M16 and PR5-1011O17 were confirmed by RT-qPCR and the lack of copy number change (both gain and loss) for RP11-100C24 as seen by FISH was also noted by RT-qPCR.

## Discussion

Our study of 27 normal subjects revealed a total of 42 CNVs: 26 previously described CNVs [14] and 16 (38%) novel CNVs. A higher number of known than novel CNVs showed recurrence in the subjects tested (7/8 recurrent clones were previously described -Table 1). Similarly, evidence of segmental duplications was found in 6/8 previously described CNVs. This confirms the observation that recurring CNVs are more prevalent in the human population, and tend to be associated with segmental duplications [7], while our novel CNVs are possibly less frequent and individual specific.

The number of CNVs detected in our controls is comparable to the number obtained by other investigators using the same commercial array-CGH and cut-off levels [17-19]. However, the number is significantly smaller than that observed by Iafrate et al [6] who reported 255 CNVs in 59 individuals. Although they used the same array-CGH platform, a different dynamic website-based analytical method was applied, instead of our fixed cut-off levels of 1.2 for duplications and 0.8 for deletions, suggesting that the analytical method used plays a significant role in the number of apparent CNVs detected among individuals. Recently, a number of studies addressed the question of global genomic variation using different approaches including tiling BAC array [7,12,20], SNP polymorphisms and oligo arrays [9-11]. The number of CNVs and chromosomal regions affected varied among studies even when the same array platform was used. For example, the BAC tiling arrays detected 3654 autosomal segmental CNVs in 95 controls [7], 913 CNVs in 270 controls [12] and 258 CNVs in 100 individuals with intellectual disability and their phenotypically normal parents [20]. The differences in the populations studied may have contributed to the observed discrepancies, however, even when the same individuals were examined using a different approach (BAC array vs. SNPs) less than half (43%) of the CNVs were detected on both platforms [12]. It is now evident that none of the existing technologies can capture all human variation.

**Figure 5:**
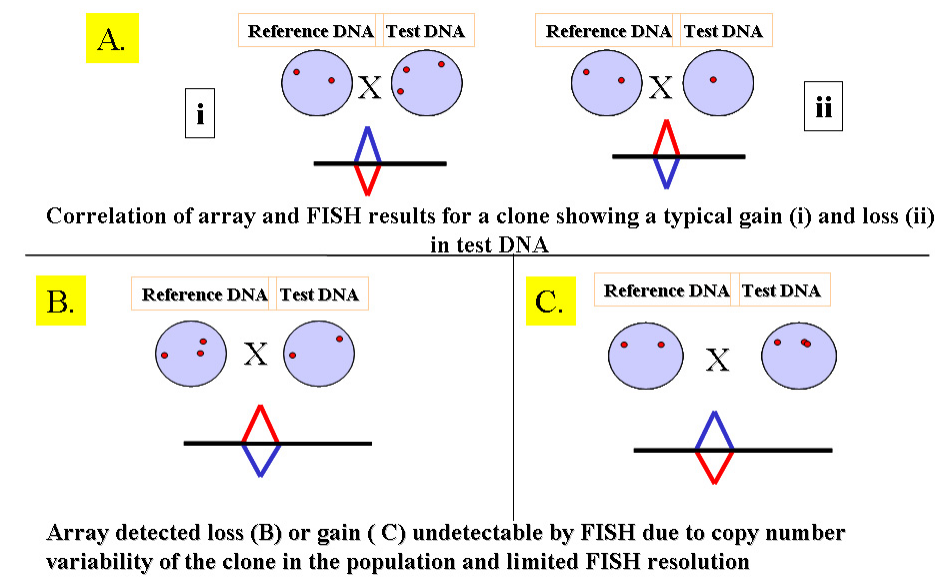
**Correlation of FISH patterns with array detected copy number variability**. The discordant results between the array and FISH/RT-qPCR findings may be due to the fact that array CGH uses the relative ratio of segmental DNA copy number in the test DNA and the reference DNA, the latter being a pool of genomic DNA from several different normal individuals. The copy number of a specific clone in the reference DNA pool determines the outcome of an array analysis (typical gain (i) and loss (ii) on the array and FISH are shown in Figure 5A). For clones with a very variable copy number, a loss on the array may simply be the result of fewer copies in the test individual compared to the pool of reference DNA (Figure 5B), and if the number of copies in the test individual is 2, confirmation by any of the methods (FISH or qPCR) may not be possible. Conversely, the gain on the array is the result of the presence of more copies of the specific DNA segment in the test DNA compared to the reference (Figure 5C). If the gain occurred as a tandem duplication (or multiplication) of the DNA segment, its detection may not be possible by FISH due to limited resolution. Alternatively, if the gain involved only some sections of the DNA segment, then it may not be detectable by RT-qPCR as typically only a small number of short sequences within non-repeated DNA segments within each region are used for analysis.

One of the pre-requisites for understanding global human variation is the confirmation of CNVs using alternative methods. Their recurrence and presence as detected using different platforms supports that these are true differences among individuals. However, a large number of CNVs are still "unique", i.e. specific for a control subject/family or study. Independent quantification methods such as FISH or RT-qPCR should ideally be performed on many CNVs, particularly those appearing in one individual, as these are the most likely ones to be false positives [7]. Considering the large number of CNVs reported (>6000 entries in the database of human variation) the number of validated CNVs using independent quantification methods such as RT-qPCR or FISH is still proportionally very small due to the time consuming or limited throughput of single locus analysis. For example, in two recent larger studies reporting a total of >5000 CNVs, less than 300 CNVs were validated using quantification methods [7,12]. Using FISH we have confirmed array-detected copy number changes of 3/6 selected CNVs (Table 2), while for 3/6 CNVs a normal two signal FISH pattern was seen. We tested two FISH-confirmed CNVs using RT-qPCR, one partially and one fully deleted (RP11-89M16 and RP5-1011O17, respectively), and observed concordance between all 3 methods (array-CGH, FISH and RT-qPCR). As two of the three FISH non-confirmed CNVs (RP11-125A5 and RP11-100C24) are recognized as being very common and recurrent on multiple platforms [6,12], we further evaluated RP11-100C24 using RT-qPCR but failed to achieve confirmation of both gain and loss. It is possible that this CNV is composed of tightly packed repeats which can be discerned only by fiber FISH, as noted for clone RP11-259N12 from chromosome 1 [6]. Additional cause of the array-CGH vs. FISH/RT-qPCR discrepancy may be due to the fact that array-CGH assays evaluate the relative ratio of segmental DNA copy number in the test DNA vs. reference DNA, and do not provide an absolute number of copies, as explained in Figure 5.

The information on new CNVs is expanding dramatically, and cataloguing clones for which independent quantification is performed are desirable, as only detailed analysis of a large number of CNVs will help better understand their basic structure, DNA content and reasons for variability. Currently, the significance of CNVs remains puzzling, as many of these genomic regions contain genes and coding sequences associated with known genetic disorders [6,8,21]. In our subjects 13/42 different CNVs were associated with OMIM genes; the number was usually not higher than 2 genes/CNV, except for CNV RP11-144O23 which had 9 genes involved in sensory perception, cell adhesion-mediated signaling, immune and defense processes (Table 1A). This clone was noted in one of our Hispanic individuals and was reported as one of the more divergent clones in the 4 populations reported by Redon et al [12]. Many of the genes in CNVs are described as "environmental sensor genes" and are associated with mechanisms mediating immune responsiveness (defensin, interferon regulatory factor 4, etc.), cellular metabolism (cytochrome P450 genes and carboxyesterase gene families), and membrane surface interactions (Rhesus blood group gene families, melanoma antigen gene). It is now established that the copy number variability of some genes can influence susceptibility to some diseases [22,23]. For example, it has been reported that people with fewer copy numbers of *CCL3L1*, a gene involved in immunity, are more susceptible to HIV infection [22]. The extent of associations of CNVs with disease susceptibility will become clearer as we learn more about the distribution of well characterized CNVs in individuals whose health and medical histories are fully evaluated.

## Conclusion

Submicroscopic CNVs are a common form of human genomic variation, which can be readily identified by array-CGH technology in phenotypically normal individuals. The number of CNVs detected in each study is influenced by several factors, especially the array platform and method of analysis. Our results confirm the wide distribution of CNVs in three different ethnic populations and expand the number of recognized CNVs. Cataloguing of confirmed CNVs, quantified using independent methods, would facilitate their interpretation and understanding of their significance in the future.

## Methods

### Subjects

Normal controls: A total of 27 normal individuals were studied. Nine Caucasian volunteers were recruited for the study and their DNA extracted and chromosomes obtained using routine methodology. Eighteen previously banked DNA samples from African-American and Hispanic individuals were also examined. All samples were anonymized for all personal identifiers.

### Array CGH

Array-CGH methods were performed as previously described [24]. Briefly, we used the commercially available genomic DNA array comprising 2,600 BAC clones with an average of 1 Mb resolution throughout the human genome (Spectral Genomics™, Houston, TX). The list of clones on this array can be obtained from website [25]. Genomic DNA from the tested subjects was extracted from peripheral venous blood using Puregene DNA Isolation Kit (Gentra Systems Inc., Minneapolis, MN, USA) according to the manufacturer's protocol. The reference DNA was purchased from Promega and represents a pool of genomic DNA from four normal control samples. Both forward (test DNA labeled with Cy3, reference DNA labeled with Cy5) and reverse labeling experiments (test DNA labeled with Cy5, reference DNA labeled with Cy3) were performed for each patient. Following hybridization, slides were scanned on a GENEPIX 4000B scanner (Axon Instruments, Union City, CA) and the 16-bit TIFF images captured using GENEPIX Pro 4.0 software. The images were analyzed using SPECTRALWARE TM BAC Array Analysis Software v2.0 (Spectral Genomics) as described previously [24]. In all cases except one, the test and reference DNA were sex matched. For the sex unmatched case, the clones on the X and Y chromosome were not considered. We have used cut-off values of 1.2 for gain and 0.8 for loss as determined previously by ourselves and others [17-19,24]. In addition, we performed one self hybridization array experiment to detect the number of artifactual gains/losses. In this latter experiment, no copy number changes were observed.

The database of human genomic variants [14] was used to check if the CNV has been previously reported and the presence of segmental duplications within it. The gene content strictly within the CNV was established using the same database as well as the NCBI and UCSC databases (build 35.1).

### FISH

BAC DNA clones that were identified to show copy number change by array-CGH were purchased from Spectral Genomics (Houston, TX), labeled directly by Spectrum Red or Green (Vysis, Downers Grove, IL) using nick translation and hybridized to metaphase chromosomes and interphase nuclei from human peripheral blood lymphocytes according to the manufacturer's instructions and as previously published [26]. Slides were viewed on a Zeiss Axioplan 2 fluorescence microscope and images captured using Macprobe software (Applied Imaging, Santa Clara, CA). For each FISH probe, at least 10 metaphase cells and 50–100 interphase nuclei were counted blindly by two observers. The normal pattern of FISH signal distribution was determined using 3 single copy BAC clones (RP1-3K23 on 7q36.3, RP11-58F7 on 7q36.3 and RP11-143E20 on Xp22.31), which showed no copy number changes in any of the control individuals on array analysis. The normal signal counts in 3 control experiments showed that most of the interphase nuclei had a concordant 1:1 and 2:2 signal pattern, while a discrepant signal number (mainly 1:2) was seen in around 20% of cells (due to asynchronous replication and/or FISH artifacts). This signal pattern is consistent with other publications using FISH with single copy clones [8].

Based on these values and our experience in FISH confirmation of microduplications [24], the predominance of cells (>50%) with a pattern different than 1:1 or 2:2 was determined arbitrarily to represent true copy number variability. Increase of DNA clone copy number was considered if a discrepant number of FISH signals (eg.1:2; 2:3), or more than 4 signals/interphase nucleus were predominantly observed (>50 % cells). A loss of the DNA clone sequences was considered if >50% interphase nuclei/metaphase chromosomes had one signal, or one of the signals was consistently fainter than the other.

### RT-qPCR

All array-detected deletions and duplications are confirmed using real-time quantitative PCR (RT-qPCR) with SYBR Green I detection [27], using 3 non-polymorphic markers evenly distributed within the deleted/duplicated clones. To ensure optimal primer design, DNA sequences spanning the target clones were retrieved from on-line sequence databases and repositories, and checked for the presence of repeated DNA sequences using RepeatMasker [28]. This allowed us to identify unique sequences within the target regions, whilst avoiding DNA segments with complex repetitive elements. Primer sets were designed within these unique sequences using the Primer Express v 3.0 program (Applied Biosystems). Primers were checked for any potential SNPs located within them using online SNP blasting.

Real-time detection of PCR products was performed using the ABI Prism 7900HT system which allows one to see the threshold cycle (C_T_) during the exponential phase of amplification (i.e. when none of the PCR reagents are limiting), and quantify each allele, such that a single allele at a test locus in a person with a deletion would show less amplification (i.e. ~50%) than in a person with two copies of that allele. We compared the amplification of test marker loci (i.e. within the region suspected of being deleted or duplicated) with that of non-contiguous markers (i.e. from another chromosomal region) performed at the same time. The list of primers used is shown in Table 3.

**Table 3:** Primers used in RT-qPCR.

**Cytoband**	**Clone name**	**Primer name**	**Forward primer (5'-3')**	**Reverse Primer (5'-3')**
2q37.3	RP5-1011O17	RP5-1011O17-A RP5-1011O17-B RP5-1011O17-C	AAATGGTGACTCTTGTGAATTTGGT GGGAAGGTGGGTGGCTACA ACACTGATGAAGGTTTTCCTTGTG	GGGAAGCTGTGGCCAAAA CAAGCAGGCCTTGGTAACACA GGCAGCACTGAACTACAGCAGTT
13q21.1	RP11-100C24	RP11-100C24-A RP11-100C24-B RP11-100C24-C	CCACCTCCCAACTCTGTGTGT CTGCTTTATGGTGCCTTTTGC TGTTTTGGCTTTCTGGCAGTT	CCCTCCAGAGATAGCACGTTCT GTCAGAGAGGACTGCGGAAACT CAAAGGCAGGAGGCTGTTCT
8p22	RP11-89M16	RP11-89M16-A RP11-89M16-B RP11-89M16-C	TTCCCAGCTCGTGCTCTCA TGGATGGTGCTAGAGAGGTAGATG CAGGATCACCCAAGGCAGTAA	CAGTGGAAGGCTCTTCATGCT TGCAGGAATGTGCTGGTTTG TCTAAACTCCCTTTTTGAGGCATT

## Authors' contributions

YQ conducted the array CGH and FISH experiments for the majority of cases, analyzed the data and drafted the manuscript. XL conducted real-time qPCR experiments. CH conducted FISH experiments, and reviewed the manuscript. SN and WTB provided DNA samples and reviewed the manuscript. MK conducted array CHG experiments and reviewed the manuscript. ERS, SL and JH designed and supervised the research study, supervised staff and students, and reviewed the manuscript. All authors read and approved the final manuscript.
